# PARP-1 Inhibition Is Neuroprotective in the R6/2 Mouse Model of Huntington’s Disease

**DOI:** 10.1371/journal.pone.0134482

**Published:** 2015-08-07

**Authors:** Antonella Cardinale, Emanuela Paldino, Carmela Giampà, Giorgio Bernardi, Francesca R. Fusco

**Affiliations:** 1 Fondazione Santa Lucia IRCCS, Laboratorio di Neuroanatomia, Roma, Italia; 2 Università Cattolica del Sacro Cuore, Dipartimento di Anatomia e Biologia, Roma, Italia; 3 Università di Roma Tor Vergata, Dipartimento di Neuroscienze, Roma, Italia; 4 Università degli studi Roma 3, Roma, Italia; Tel-Aviv University, ISRAEL

## Abstract

Poly (ADP-ribose) polymerase 1 (PARP-1) is a nuclear enzyme that is involved in physiological processes as DNA repair, genomic stability, and apoptosis. Moreover, published studies demonstrated that PARP-1 mediates necrotic cell death in response to excessive DNA damage under certain pathological conditions. In Huntington’s disease brains, PARP immunoreactivity was described in neurons and in glial cells, thereby suggesting the involvement of apoptosis in HD. In this study, we sought to determine if the PARP-1 inhibitor exerts a neuroprotective effect in R6/2 mutant mice, which recapitulates, in many aspects, human HD. Transgenic mice were treated with the PARP-1 inhibitor INO-1001 mg/Kg daily starting from 4 weeks of age. After transcardial perfusion, histological and immunohistochemical studies were performed. We found that INO 1001-treated R6/2 mice survived longer and displayed less severe signs of neurological dysfunction than the vehicle treated ones. Primary outcome measures such as striatal atrophy, morphology of striatal neurons, neuronal intranuclear inclusions and microglial reaction confirmed a neuroprotective effect of the compound. INO-1001 was effective in significantly increasing activated CREB and BDNF in the striatal spiny neurons, which might account for the beneficial effects observed in this model. Our findings show that PARP-1 inhibition could be considered as a valid therapeutic approach for HD.

## Introduction

Huntington’s disease (HD) is a severe neurodegenerative disorder, genetically transmitted in an autosomal dominant fashion, and it is characterized by motor dysfunction, cognitive decline and psychiatric disorder [1–2]. The IT15 gene [3] encoding for huntingtin protein consists in a CAG expansion beyond the normal 10–35 repeat range [4]. HD pathology is characterized by the formation of neuronal intranuclear inclusions that are constituted by mutated huntingtin [5]. Such inclusions interact with and impair several cellular functions [6]. Apoptosis gives rise to the removal of damaged or unnecessary cells, and this phenomenon occurs physiologically during development or after DNA damage and normal tissue homeostasis [7, 8, 9]. The activation of unharmonious apoptosis has been implicated in HD, as shown by several studies [10, 11]. Indeed, several studies showed apoptotic cells and DNA degradation products both in HD patients’ brains [11, 12, 13, 14] and in experimental models of HD [15, 16, 17]. In particular, Vis and coworkers [11] described Poly (ADP-ribose) polymerase (PARP) expression and TUNEL labeling in HD brains. PARP is a ubiquitous enzyme connected to DNA repair and associated with many cellular functions such as preservation of genomic stability and cell death. PARP converts nicotinamide adenine dinucleotide (NAD) into poly (ADP-ribose) (PAR) polymers binding them to several different DNA binding proteins. Excessive activation of PARP induced by DNA damage depletes intracellular NAD stores and leads to cell death [18, 19].

An over activation of PARP has been associated with the pathogenesis of several nervous system disorders, such as excitotoxicity, traumatic injury, ischemia-reperfusion and inflammation [20, 21, 22, 23, 24].

A disproportionate activation of PARP evokes the release of apoptosis-inducing factor (AIF) [25, 26]. PARP also leads to the translocation of AIF from the mitochondria to the nucleus and causes a programmed cell death named parthanatos [27]. Such mechanism is not related to caspase. Inhibition of PARP by specific compounds can prevent the release of AIF to the nucleus and therefore is protective for the cell [27, 28].

On the other hand, oxygen and glucose depletion that occurs in conditions of acute ischemia, such as stroke and myocardial infarction, is responsible for an excessive release of reactive oxygen species (ROS) and nitric oxide [29]. This phenomenon leads to DNA damage, and therefore to PARP activation [30, 31], which in turn leads to NAD+ and ATP depletion. Decrease in ATP is the cause of DNA damage. Excessive activation of PARP by stress stimuli, such as ROS formation, has been associated with the pathogenesis of several diseases, such Parkinson’s disease [32, 33], ischemia-reperfusion—induced cardiac dysfunction [34, 35], diabetic complications [36]. Therefore, PARP inhibitors can be used to prevent oxidative stress-induced cell death.

Recently, it was demonstrated that PARP inhibitors could restore sensitivity of resistant tumors to standard chemotherapy [37]. Interestingly, PARP inhibitors may also provide protection from the unanticipated effects of chemotherapy agents, which cause oxidative stress and, consequently, PARP overactivation. Moreover, PARP inhibition has been postulated to exert a neuroprotective action in neurodegeneration induced by acute brain ischemia [38].

In this study, we have explored the possibility that PARP inhibition could be effective in achieving neuroprotection in the transgenic model of Huntington’s disease.

It has been established that mutant huntingtin impairs cAMP signaling [39,40] and gene transcription mediated by the cAMP response element–binding protein (CREB) [41]. Inhibition of CREB-mediated transcription is involved in the neurodegeneration occurring in HD [42] and a decreased transcription of CREB-regulated genes is observed in HD transgenic animals [43].

Thus, counteracting the decreased cAMP signaling and loss of CREB-regulated transcription may be beneficial in treating HD neurodegeneration [44].

One of the key downstream mediators of CREB signaling cascade is BDNF, a principal neurotrophic factor for both striatal and cortical neurons. BDNF is greatly diminished in HD.

Thus, we investigated the effects of PARP 1 inhibition on CREB phosphorylation (pCREB) and BDNF in the R6/2 model of HD.

## Materials and Methods

### Animals and drug administration

All studies were conducted in accordance with European Communities Council Directive of 24 November 1986 (86/609/EEC) as adopted by the Santa Lucia Foundation Animal Care and Use committee. The Santa Lucia Foundation Animal Care and Use committee specifically approved this study.

Transgenic R6/2 strain was obtained by ovarian transplant of hemi zygote females x B6CBAF1/J males, all obtained from Jackson Laboratories (Bar Harbor, ME). All the experiments were conducted on F1 mice. The offspring were genotyped by PCR assay of DNA obtained from tail tissue. After genotyping at 4 weeks of age, mice were weaned and the treatments started. Twenty-four mice per group were used. The study groups included: R6/2 mice that were given 0.9% saline by intraperitoneal (i.p.) injection, R6/2 mice with INO-1001 given by i.p. injection dissolved in saline (10 mg/kg/day), INO-1001 treated wild type mice and saline-treated wild-type mice. Mice were handled under the same conditions by one investigator at the same day and time. Mice were identified by a randomly assigned code. Mice were housed five in each cage under standard conditions with ad libitum access to food and water.

Data were collected by observers who were blinded to treatment.

### Survival

For the survival study, the criterion for euthanasia was the point when animals were not able to right themselves after 30 s when placed on their side, according to Stack and co-workers [45].

### Weight

Animals were weighed weekly starting at the beginning of treatment and until the time of sacrifice. Their weight was recorded, plotted, and percentage change on body weight was calculated according to the following formula: body weight on sacrifice day—body weight on day 35/body weight on day 35 [46]. Day 35 represents the first day of the 5^th^ week from the beginning of treatment.

### Behavior

#### 1. Assessment of neurological abnormality

Mice of the R6/2 strain exhibit a hind-limb clasping phenotype when suspended by the tail [1–47]. The clasping phenotype has been extensively studied and used to recognize the neurological impairment in HD mice [48, 49], and it is considered a measure of disease progression [50]. Mice were suspended by their tail for 60 seconds. The total amount of time spent clasping the hind limb was recorded twice weekly.

#### 2. Rotarod

The five-station rotarod performance test (Rotarod/RS LSI Letica, Biological Instruments, Varese, Italy) was used to estimate mice motor coordination and balance. During the test, mice were placed on a horizontally rotating rod, which is down enough to prevent animal damages, but high enough to induce the fall. Mice started Rotarod performance test twice weekly from five to fourteen weeks of age. Three trials measurements on the rod for their latency or fall were recorded. A maximum latency of 60 sec was defined for mice that did not fall.

#### 3. Open field

Motor activity and anxiety were measured in an open field consisting in a circular arena with a 60 cm diameter, white floor and divided into central and peripheral areas by drawing black line. The open field measurements were performed in a soundproof room illuminated by an 80W red ceiling light. The video camera on the arena was connected to a video recorder of a computer placed in the next room. Mice were placed into the arena for 10 minutes, while distance traveled and velocity were recorded and analyzed by a specific software (Noldus, Wageningen, the Netherlands).

### Histological and immunohistochemical studies: Tissue processing

For the histological examination, mice were transcardially perfused under deep anesthesia, with saline solution containing 0.01 ml heparin, followed by 60 ml of 4% paraformaldehyde in saline solution. Brains were removed, collected and post fixed overnight at +4°C, then cryoprotected in 10% sucrose and 20% glycerol in 0.1M phosphate buffer (PB) with sodium azide 0.02% for 48 h at 4°C. Brains were subsequently sectioned frozen on a sliding microtome at 40 μm thickness to obtain serial sections.

#### Immunohistochemistry

For the immunohistochemical studies, primary omission controls, normal mouse and rabbit serum controls and preimmune serum controls were used to confirm the specificity of our immunohistochemical labeling.

#### Neuropathological (primary) outcome measures

1. Evaluation of gross striatal volume. Standard Nissl staining was employed on coronal step serial sections from rostral neostriatum through the level of anterior commissure (interaural 4.66 mm/bregma 0.86 mm to interaural 3.34 mm/bregma -0.46 mm) from six animals per group. Gross striatal volume was measured using Neurolucida Stereo Investigator software (Zeiss, Cochester, VT, USA).
2. Striatal neuronal cell count. Single label immunofluorescence was performed using an antibody against Calbindin D-28K (marker of the Medium Spiny Neurons) (CALB, Immunological Sciences) to evaluate the number of surviving projection neurons in the striatum. Cell count were carried out in each of 19 1.0-mm-square confocal microscope fields, rostrocaudally spaced on both hemispheres of 6 mice from each group. For cell counts, we used Java image processing and analysis program Image J, developed by Wayne Rasband, available in the public domain (http://imagej.nih.gov/ij/docs/index.html). We used a manual approach, measuring the number of objects by means of the point selection tool.
3. Microglial morphology. Microglial morphology was studied by immunolabeling our tissue with an antibody for microglia (mouse anti-CD-11b, Serotec). The diaminobenzidine–immunoperoxidase technique was employed as previously described [51].
4. Evaluation of NIIs. Sections were processed for single label EM-48 mutant huntingtin protein antibody (Clone mEM48, Millipore Corporation) by means of immunofluorescence, and were counterstained with NeuroTrace. A sample of about 250 neurons per hemisphere for each of three sections in each of 6 mice per treatment group was analyzed to determine the number of NIIs of striatal neurons in both saline and INO-1001 treated R6/2 mice (wild type littermates did not show NIIs–like mutant huntingtin immunoreactivity- data not shown).

#### Double label immunohistochemical studies: Protein expression of CREB and BDNF

1. Analysis of CREB activation in the surviving striatal spiny neurons. We employed a dual label immunofluorescence technique with antibodies directed against calbindin and pCREB, respectively, in order to identify the intensity of phosphorylated CREB in the striatal spiny projection neurons [52]. Tissue was mounted on gelatin-coated slides, cover slipped with GEL-MOUNT and examined under an epi-illumination fluorescence microscope (Zeiss Axioskop 2). A confocal laser scanner microscopy (Zeiss LSM 700) was subsequently used to acquire all the images for quantification. Quantification was carried out by using the Java image processing and analysis program, Image J (see above) [53, 54]. Briefly, we selected cells of interest using a circle selection tool. From the Analyze Menu→Set measurement we selected “Mean Grey Value”, “Area” and “Min&Max Grey Value”. The region next to cells with no fluorescence was considered “*background*” and subtracted. Finally, the “Measure” tool was selected from the Analyze menu and a mean value was obtained.
2. Analysis of Brain Derived Neurotrophic Factor (BDNF) in the striatal spiny neurons. To study changes in BDNF in the striatal projection neurons after INO-1001 administration, double label immunofluorescence was employed using an antibody against BDNF (mouse anti-BDNF, Immunological Sciences, Italy) combined to anti-calbindin antibody [55].

To evaluate the intensity BDNF immunolabeling, an image analysis of BDNF immunoreactive projection neurons was performed as described above. The intensity of BDNF immunoreactivity per field, expressed in arbitrary units, was calculated by the Image J software (see above) and a mean value was obtained.

#### Immunohistochemical studies: iNOS expression

To study iNOS expression, we performed a single-labeled immunofluorescence, using an antibody against iNOS (rabbit anti-iNOS-Immunological Science) counterstained with NeuroTrace fluorescent Nissl (visualized by green fluorescence). Antigen retrieval was performed in Citrate Buffer (pH 6–7) for 20 minutes at 80°C. Subsequently, sections were incubated rabbit anti-iNOS antibody (1:100) for 72 hours at +4°C. A streptavidin–biotin amplification method for iNOS immunofluorescence staining was used.

### Western Blotting

Western blot analysis was performed in R62 mice treated with vehicle or INO-1001, and wild type mice were used as control. Striatal protein concentrations were determined with a BCA Assay Kit (Pierce, Rochford, IL, USA). Equal amounts of protein were separated by SDS-polyacrylamide gel electrophoresis and transferred onto PVDF membranes. The membranes were blocked using nonfat milk in Tris-buffered saline (TBS)/Tween-20 and then incubated with antibodies against Bcl2 (rabbit 1:500, Immunological Science) and Bax (rabbit 1:500, Immunological Science) at +4°C overnight. After being washed with TBST, the membranes were incubated with an HRP-labeled secondary antibody, developed using an ECL Detection Kit (Santa Cruz biotechnology, Inc) and analyzed using Image J software. Striatal samples for western blot analysis were collected from four mice.

### Statistical analysis

The data collected were analyzed to compare the effect of INO-1001 on weight, clasping, and on surviving cell number, NIIs percentage, BDNF expression and CREB activation in the striatal of differently treated groups. Statistical analysis was performed by STATISTICA 10 software and ANOVA available on the software GraphPad Prism version 6.0. *P* values of less than 0.05 were considered statistically significant. Survival data were analyzed by means of a product limit method of Kaplan and Meier, and P value was set at <0.0001 for significant results.

## Results

### Survival

**Fig 1:**
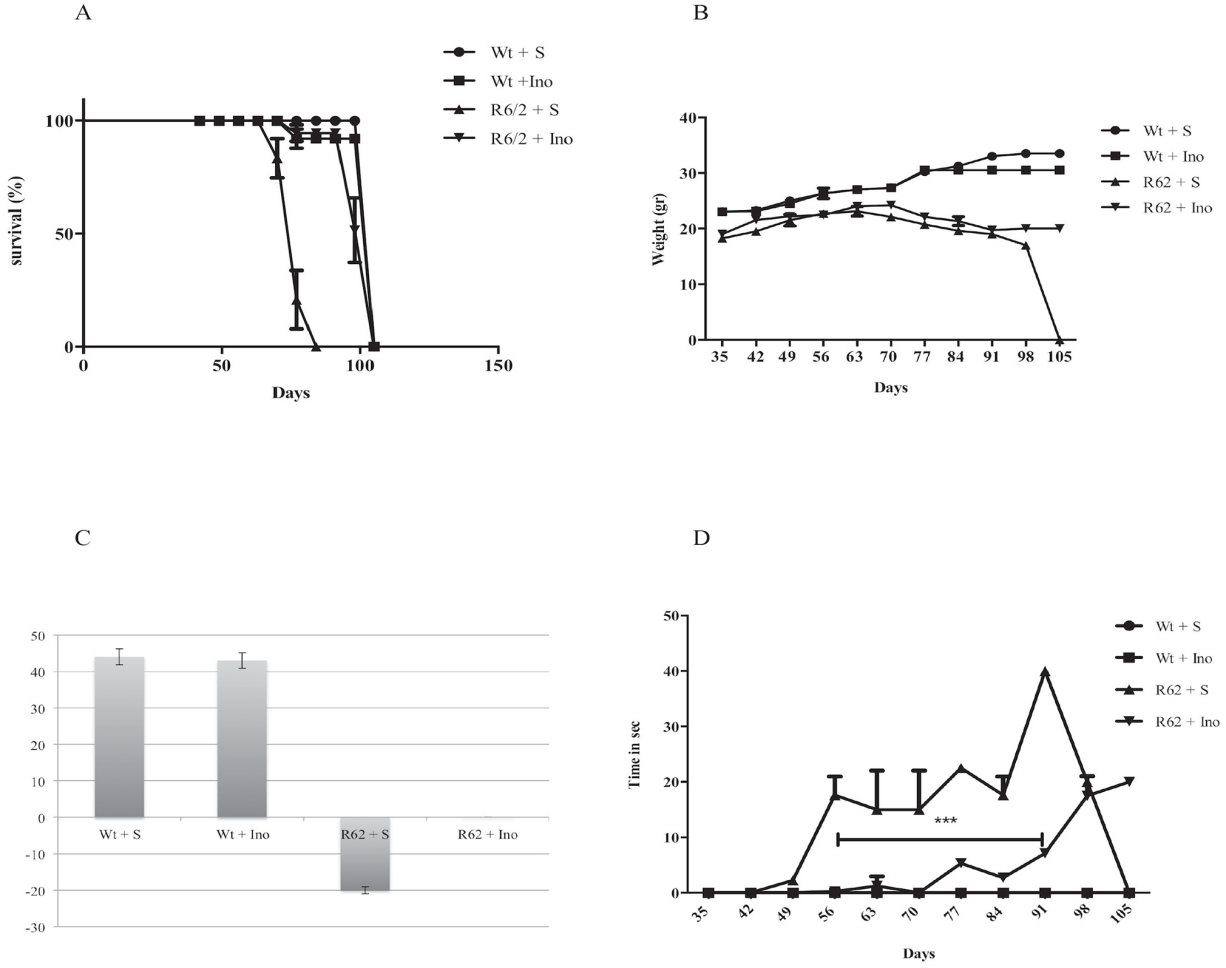
In (**A**) the survival is described by means of a Kaplan-Meier curve. R6/2 mice treated with INO-1001 showed a mean survival time that was significantly (*P*<0,0001) longer than that of R6/2 mice treated with saline. (**B**) INO-1001 effects on wild type and R6/2 mice weight. A three-way ANOVA shows a statistically significant effect of genotype (F(1,18) = 321,16;*P*<0,0001),treatment(F(1,18) = 9,02;*P*<0,0076),time(F(9,162) = 27,74;*P*<0,0001) and a genotype X treatment X time interaction (F(9,162) = 7,52;*P*<0,0001) in R6/2 mice treated with INO-1001(**C**). Hind-limb clasping phenotype in R6/2 mice treated with INO-1001. Data analysis indicates a statistically significant effect of treatment and age. INO-1001 treated mice showed significantly less clasping at week 10,11,12 and 13 (**D**)(*** *P*<0,001).

The treatment with INO-1001 proved beneficial for R6/2 mice as shown by the Kaplan Meier survival curve. In our study, R6/2 mice were followed at every time point (weeks) until their death. Saline treated R6/2 mice, expressed as a percentage, died approximately at day 80, but the other groups, wild type and INO-1001 treated mice survived for more days or until the time they died as it is showed in the graph (Fig 1A).

### Weight

Weights among different treatment groups’ mice changed during the time course. The differences were not significant until 10 weeks. A graph describing all the weight changes among the different groups is shown in Fig 1B. At 11 weeks, wild type saline treated animals weighed 31+/-0.25g. The differences were not statistically significant with INO-1001 treated wild type animals. INO-1001 treated R6/2 mice weighed 23 +/-1.35, whereas saline treated R6/2 mice weighed 17+/-0.75g. In Fig 1C we show that R6/2 treated with saline lost weight significantly, whereas INO-1001 treated R6/2 weight loss was not significant.

### Assessment of neurological abnormality

Paw clasping occurred only after 8 weeks of age. INO-1001 ameliorated the progression of neurological abnormality in the R6/2 mice. No clasping activity was present in the wild type mice. The time spent clasping was significantly less in the INO-1001 treated group than in the saline group, as shown in Fig 1D (P<0.001).

### Rotarod

**Fig 2:**
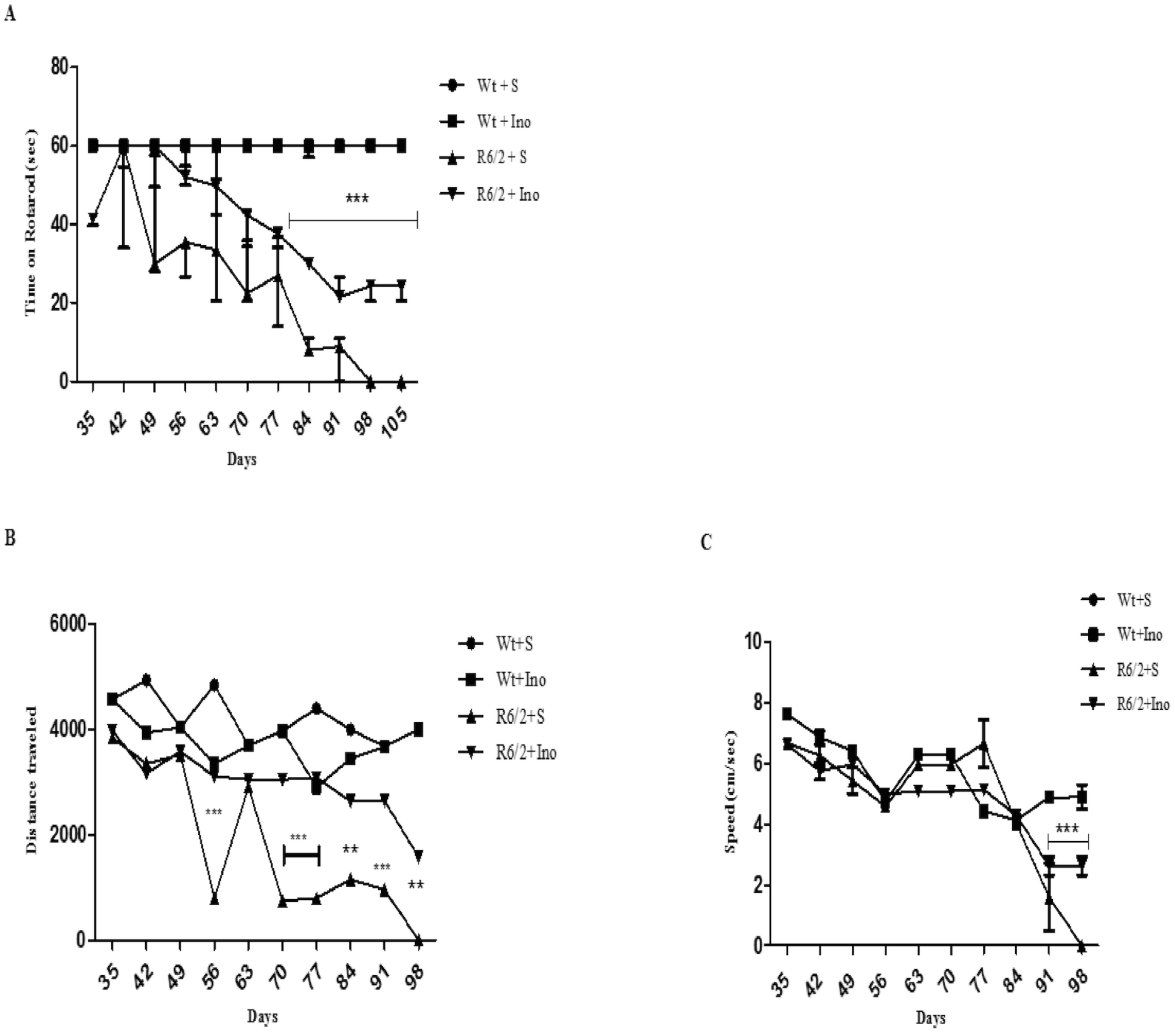
Effect of INO-1001 treatment on motor coordination. A: Graph shows values as mean time SEM spent on Rotarod by 15-week-old wild type mice and R6/2 mice treated with INO-1001 and saline. A three-way ANOVA with genotype, treatment and time as main factors, revealed that R6/2 mice had a statistically significant impairment in motor coordination compared to wild type mice F(1,18) = 90,28; p<0,0001 and that INO-treatment improved performance in a genotype-dependent fashion F(1,18) = 11,28; p<0,0035. Moreover, Post hoc analysis shows no differences in motor performances between INO-1001-treated and saline-treated wild type mice, ruling out any toxic effect of INO-1001. B and C: Effect of INO-1001 treatment on motor activity in the open field task. Values represent mean SEM for the distance traveled in the arena and speed by 14 week old wild type mice and R6/2 mice treated with saline or INO 1001. Open field data at 15^th^ week were not be acquired. R6/2 mice traveled a shorter distance at a lower speed than wild type mice (genotype effect with F (1, 18) = 54, 79; p<0,0001). Instead, INO-1001 treatment was able to promote the rescue of motor performances in a genotype dependent fashion (significant genotype X treatment interaction F (1, 18) = 6, 04; p<0,0244).

A three-way ANOVA with genotype, treatment and time as main factors, revealed that R6/2 mice had a statistically significant impairment in motor coordination compared to wild type mice F(1,18) = 90,28; p<0,0001 and that INO-treatment improved performance in a genotype-dependent fashion F(1,18) = 11,28; p<0,0035. Moreover, Post hoc analysis showed no differences in motor performances between INO-1001-treated and saline-treated wild type mice, thereby ruling out any toxic effect of INO-1001 (Fig 2A).

### Open Field

Fig 2B and 2C show motor activity data collected in the open field task, including the total distance traveled and speed of locomotion in the arena. R6/2 mice traveled a shorter distance at a lower speed than wild type mice (genotype effect with F (1, 18) = 54, 79; p<0,0001). Instead, INO-1001 treatment was able to promote the rescue of motor performances in a genotype dependent fashion (significant genotype X treatment interaction F (1, 18) = 6, 04; p<0,0244).

### Neuropathological outcome measures

#### 1) Striatal volume

**Fig 3:**
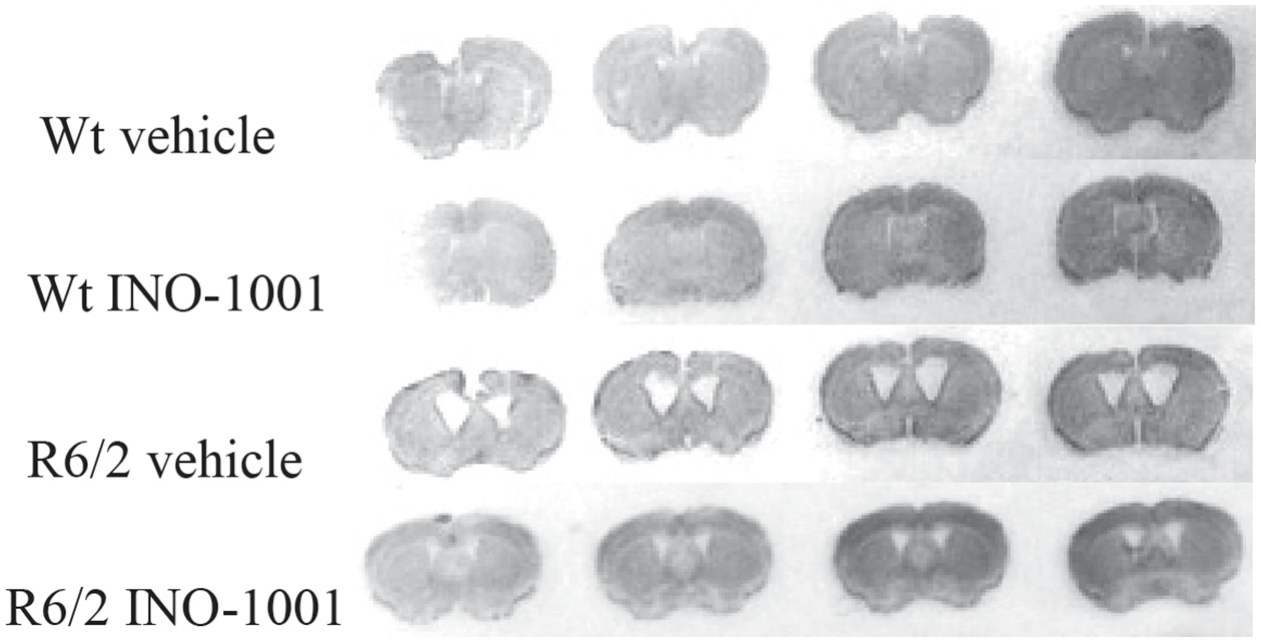
Effects of INO-1001 treatment on striatal atrophy in R6/2 mice. Transmitted light microscope images showing Nissl-stained coronal sections of wild-type mouse, vehicle treated wild-type mouse, vehicle treated R6/2 mouse and INO-1001-treated R6/2 mouse. R6/2 mice treated with vehicle display marked gross atrophy and enlarged ventricles compared to R6/2 mice treated with INO-1001.

The gross striatal volume of saline treated R6/2 mice was greatly reduced compared to their wild type littermates (Fig 3), in agreement with other studies [45, 56].

**Fig 4:**
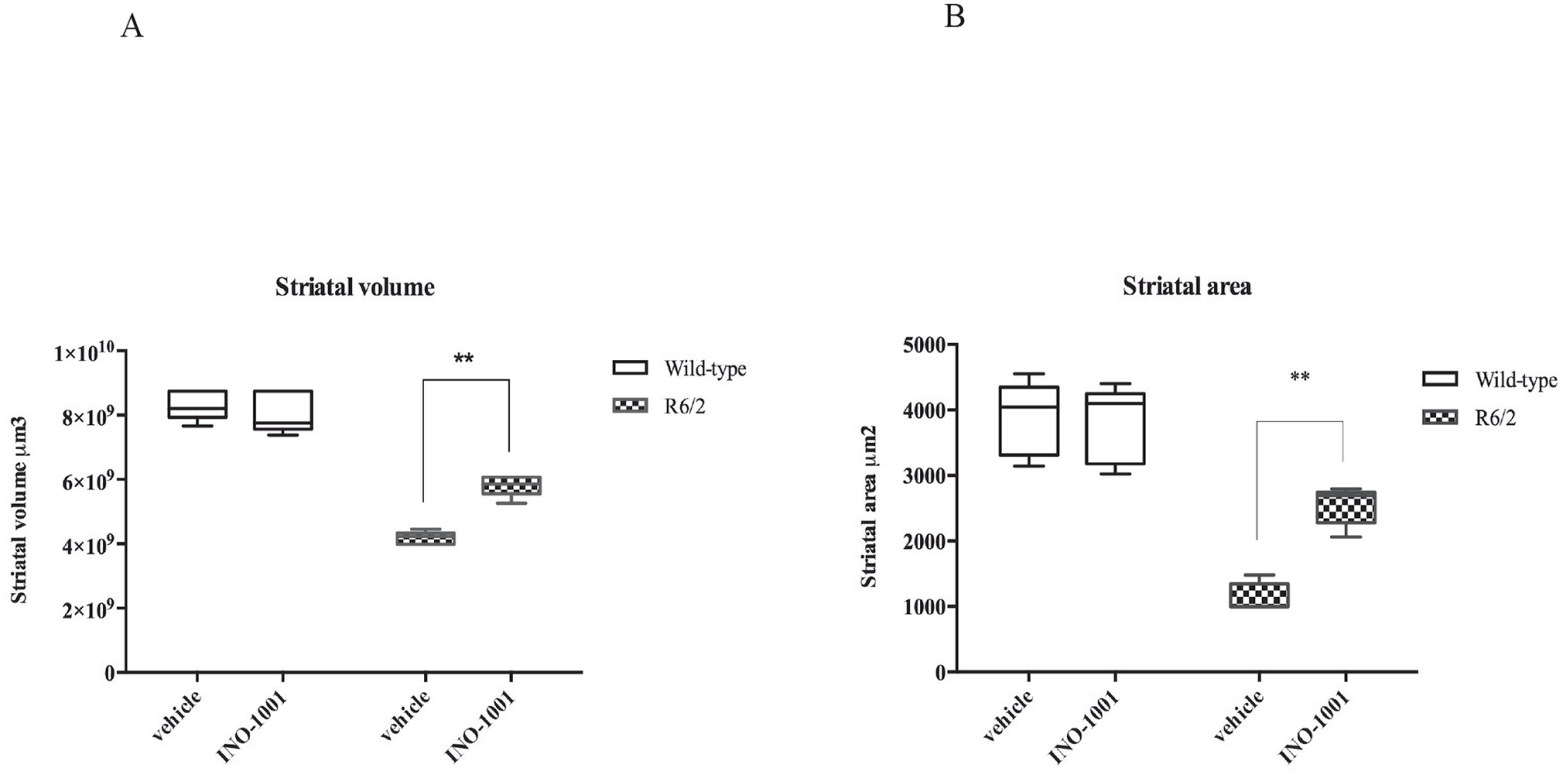
Quantification of differences in striatal volume. **(A)** Two-way ANOVA indicated a statistically significant effect of genotype (F(1,16) = 272,6;*P*<0,0001),treatment(F(1,16) = 13,29;*P*<0,0022) and genotype X treatment interaction in mice treated with INO-1001 (F(1,16) = 23,70;*P*<0,0022). (B) Quantification of differences in striatal area. R6/2 treated with vehicle had a statistically significant reduced striatal area compared to wild type. Striatal area of R6/2 mice treated with INO-1001 was significantly greater than that of vehicle treated R6/2 mice (** *P*<0,0040).

The mean striatal volume was 4.18+/-0.27 X 107 μm3 in the saline treated R6/2 mice, whereas it was 8.31+/-0.21 x 107 μm3 in the wild type animals. The striatal volume of INO-1001 treated R6/2 mice was 5.92+/-0.20 x 107 μm3 (*P*<0.002). INO-1001 attenuated striatal atrophy of R6/2 mice, as shown in Fig 4A.

#### 2) Striatal area

Our histological observations showed that the striatal area of saline treated R6/2 mice was smaller than wild type animals (with an average area of 1000 μm2 compared to 4554 μm2 of the wild type littermates). Treatment of R6/2 mice with INO-1001 reduced the loss of striatal size in a statistically significant fashion (2794.98+/-0.41 x 105 μm2) as shown in Fig 4B (*P*<0.004).

#### 3) Striatal neuronal cell count

**Fig 5:**
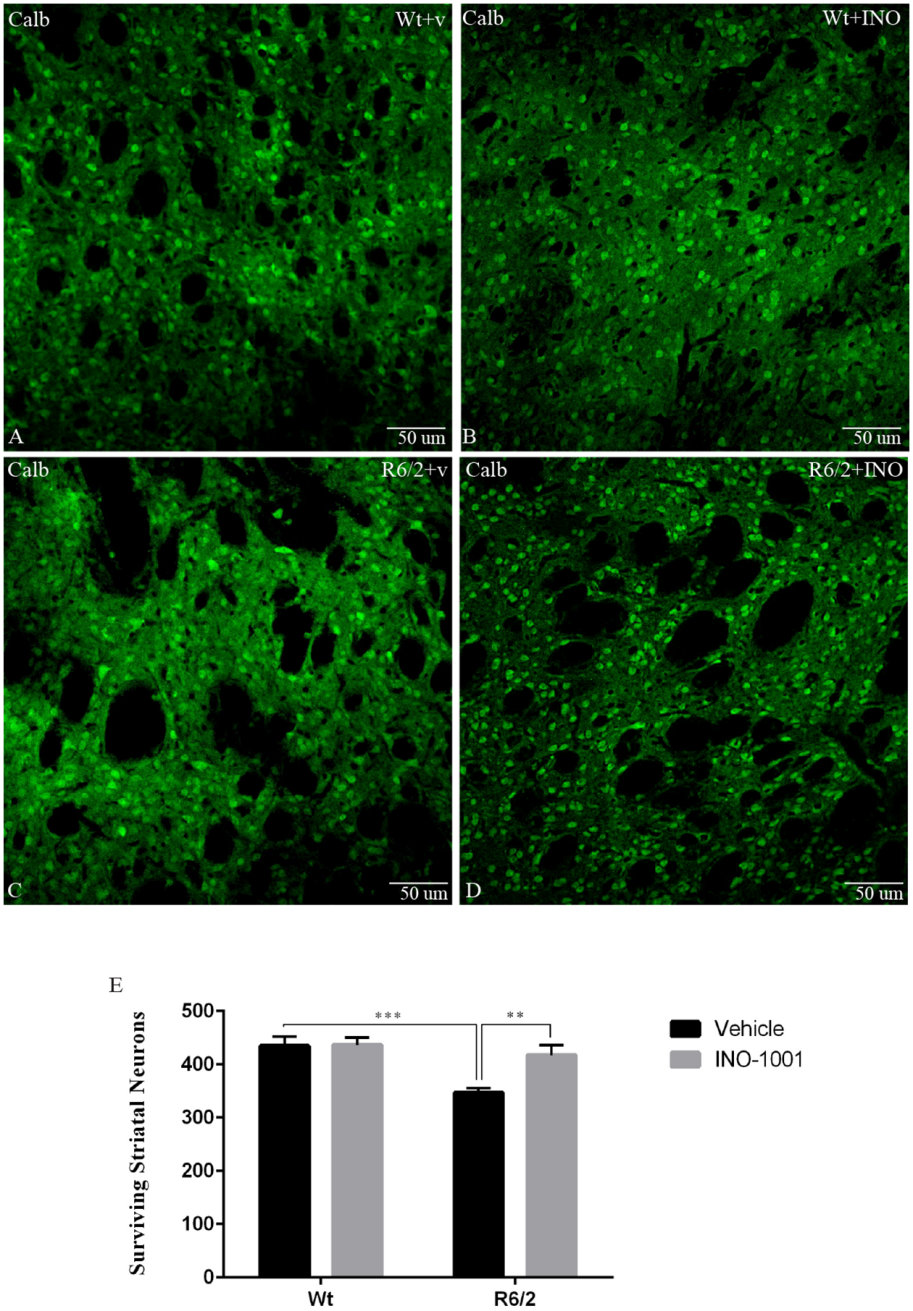
Effects of INO-1001 treatment on striatal neuron number in R6/2 mice. Confocal laser scanning microscopy images of single-label immunofluorescence for the marker of the medium spiny neurons, Calbindin D-28K (green), in the striatum of a vehicle treated wild type (**A**), INO-1001 treated wild-type (**B**), vehicle treated R6/2(**C**), or R6/2 mouse treated with INO-1001 from 5 weeks of age (**D**). (**E**) Quantification of the number of striatal neurons labeled with CALB. A two way ANOVA indicated a significant effect of genotype (F(1,72) = 12,18474; p = 0,0008), treatment (F(1,72) = 5,442040; p = 0,0225), and genotype X treatment interaction (F(1,72) = 5,019067; p = 0,0282). Data were analyzed with GraphPad Prism statistical software 6.0e using two-way ANOVA followed by a Tukey’s multiple comparison test. A Tukey’s multiple comparison test indicated that the density of striatal neurons in R6/2 mice treated with INO-1001 was significantly greater than that of vehicle treated R6/2 animals (*P*<0.001).

In the wild type mice, the average number of CALB positive cells per mm^2^ was 434, 89 +/- 75, 7, which was significantly reduced to 346, 84 +/- 39, 03 in the vehicle-treated R6/2 mice (Fig 5). INO-1001 treatment almost completely prevented the decrease in CALB-positive neurons (417, 1 +/- 82, 01 CALB-positive neurons per microscope field) in the R6/2 mice. Moreover, the two-way ANOVA analysis confirmed that there was a statistically significant difference in MSN number. In fact, Tukey’s multiple comparison test showed that neurons density in vehicle-treated R6/2 mice was lower than wild type mice (vehicle or INO-1001) or the INO-1001-treated R6/2 mice.

### Histological and immunohistochemical studies

#### 1) Study of the microglia

**Fig 6:**
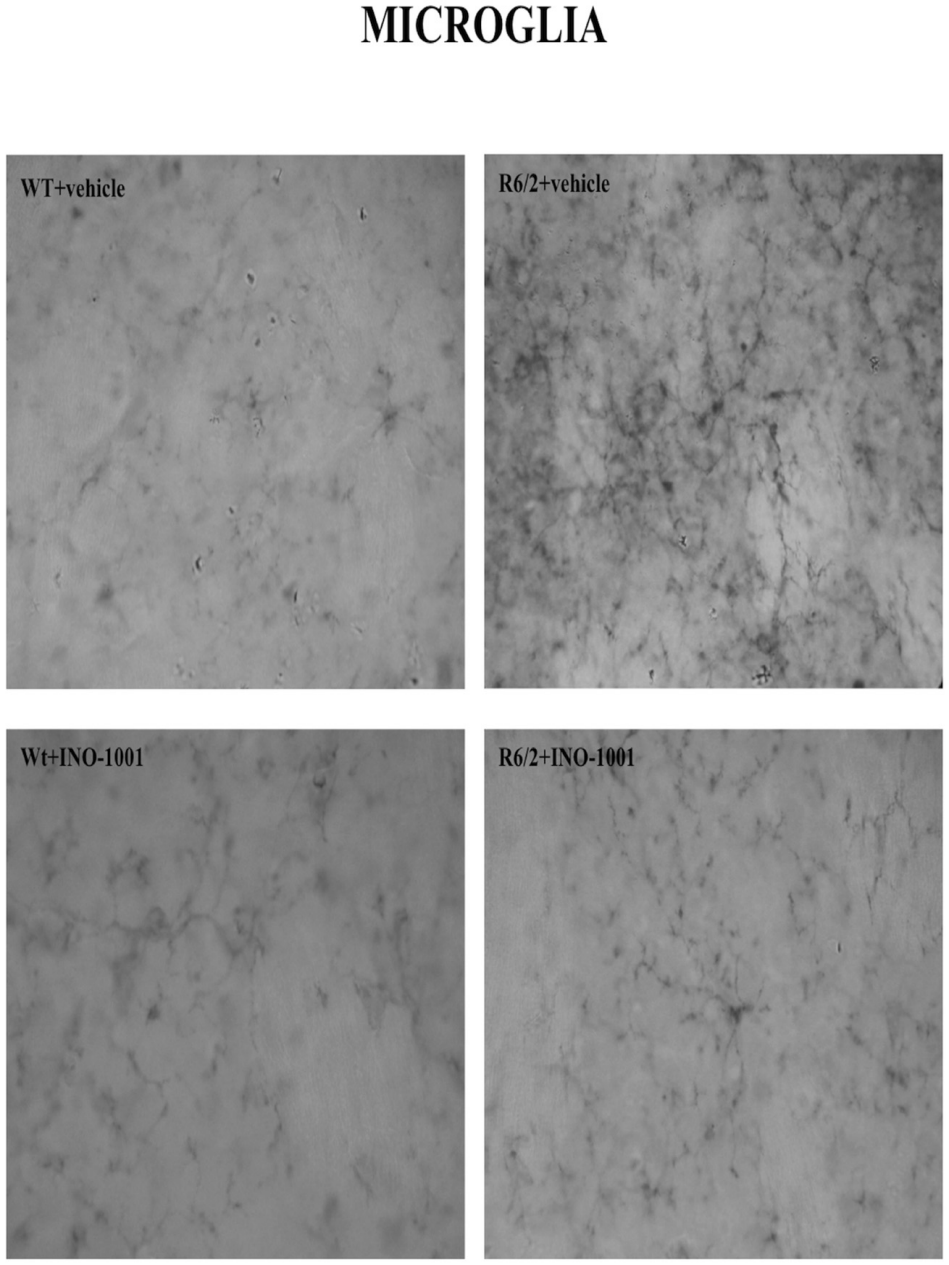
Effects of INO-1001 treatment on reactive microglia. Representative photomicrographs of DAB immunohistochemistry for the marker of activated microglia, CD11b, in the striatum of a vehicle treated wild type (**A**), INO-1001 treated wild-type (**B**), vehicle treated R6/2(**C**), or R6/2 mouse treated with INO-1001 from 5 weeks of age (**D**). In the sample from the vehicle treated R6/2 mouse, there is an apparent intense microglial reaction, in which microglial cells appear numerous and with coarse arborizations and a rod-shaped body. In the sample from a INO-1001 treated R6/2 mouse, there are fewer reactive cells, along with quiescent cells.

CD-11b immunohistochemistry was performed for the detection of microglia. Wild type mice treated with saline and with INO-1001 displayed scattered, quiescent microglia, with no cellular processes and an ovoid body. On the contrary, immunostaining for CD-11b in the saline treated R6/2 group revealed an intense microglial reaction, where microglial cells appeared numerous and displayed coarse arborizations. Microglial reaction appeared markedly attenuated in INO-1001 treated R6/2 mice (Fig 6).

#### 2) Study of NIIs in the striatum

**Fig 7:**
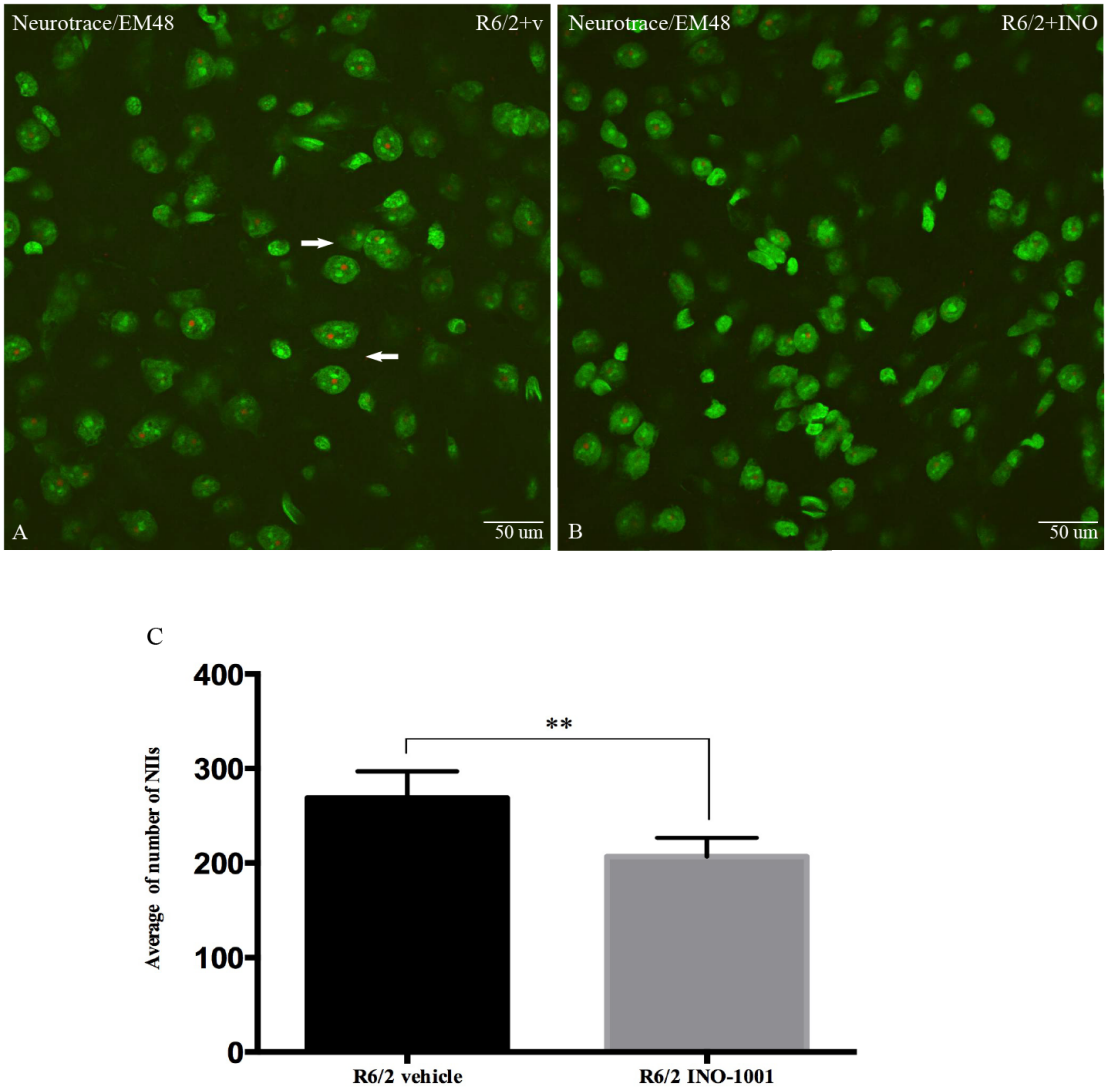
Effects of INO-1001 treatment on the density of NIIs. Confocal laser scanning microscopy images of single-label immunofluorescence for NIIs marker EM48 (red), which labels neuronal intranuclear inclusions containing aggregated mutant huntingtin (NIIs) in the striatum of vehicle treated R6/2 (**A**), or R6/2 mice treated with INO-1001 (**B**) from 5 weeks of age and counterstained with NeuroTrace fluorescent Nissl (visualized by green fluorescence). (**E**) Quantification of NIIs in vehicle-or-INO-1001 treated R6/2 mice. There were no NIIs detected in striatum of vehicle-or-INO-1001 treated wild-type mice, so this group was not included in the statistical analysis. A t-test indicated that the density of NIIs in striatum of R6/2 mice treated with INO-1001 was lower than that in R6/2 mice treated with vehicle (*P*<0.05 = 0.0026). Please note the arrow indicates a neuronal cell body with NIIs in the R6/2 mouse treated with saline.

In this part of the study, we investigated whether INO-1001 treatment affected NIIs formation in the striatum. No NIIs (as immunolabeled for EM-48 mutant huntingtin) were observed in the wild type animals (Figures not shown). By analyzing EM-48 immunohistochemistry in sections counterstained with NeuroTrace, we observed that the number of NIIs in the striatal neurons of R6/2 mice treated with saline was significantly higher compared to the INO-1001 treated group (Fig 7A and 7B). Thus, a marked reduction in the number of NIIs demonstrated that INO-1001 treatment delayed the development of inclusions in R6/2 mice.

#### 3) Analysis of CREB activation in the surviving striatal spiny neurons

**Fig 8:**
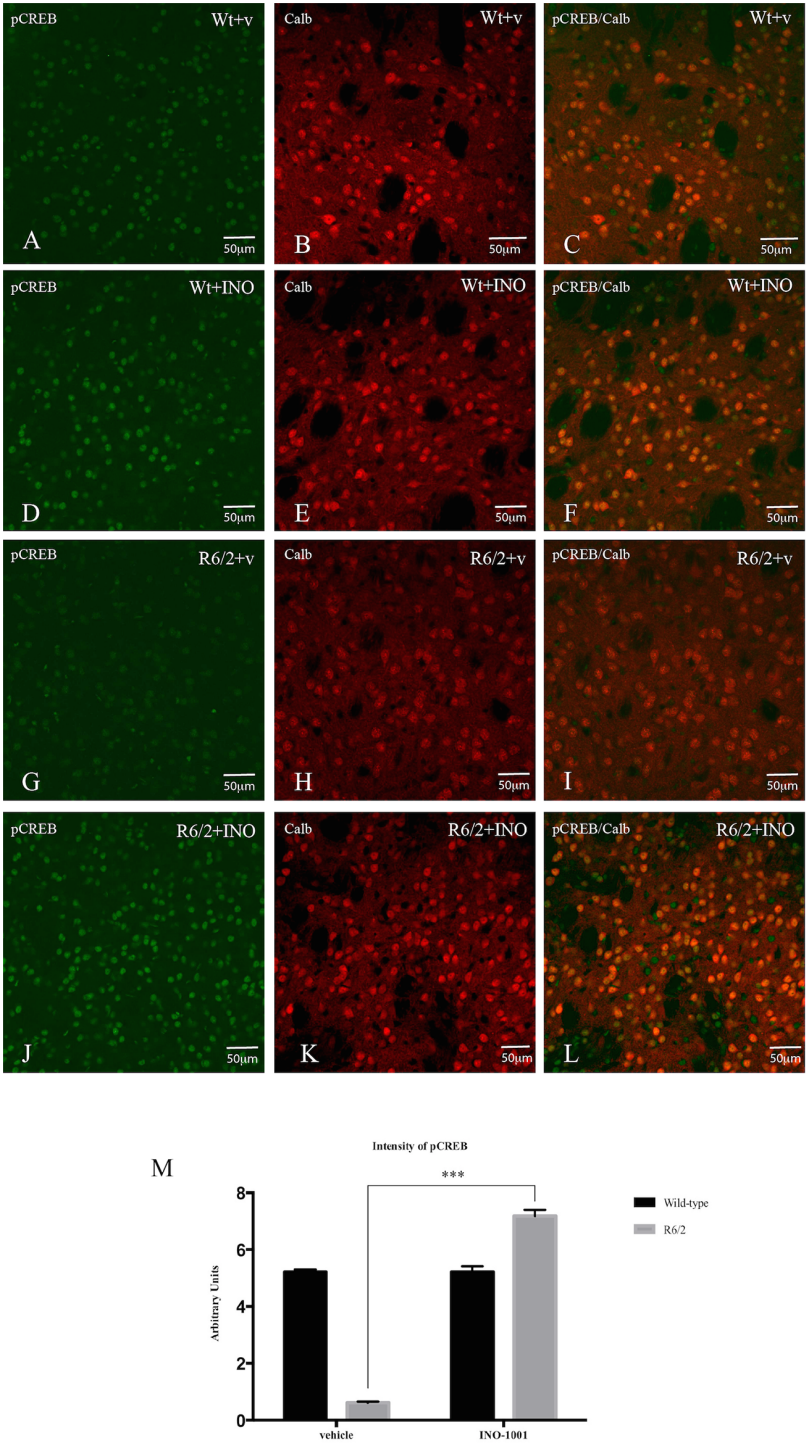
Effects of INO-1001 treatment on CREB activation. Representative confocal laser scanning microscopy images of dual label immunofluorescence for CALB (red) and pCREB (green) in striatal samples from a vehicle treated wild type (**A-B-C**), INO-1001 treated wild type (**D-E-F**), vehicle treated R6/2 (**G-H-I**) or R6/2 treated with INO-1001 mouse (**J-K-L**). (**M**) Quantification of the intensity of pCREB immunoreactivity associated with CALB-labeled striatal neurons. A two way ANOVA indicated a significant effect of genotype, treatment and genotype X treatment interaction (please see main text). Data were analyzed with GraphPad Prism statistical software 6.0e using two-way ANOVA followed by a Tukey’s multiple comparison test. A Tukey’s multiple comparison test indicated that there was no statistically significant difference in pCREB level wild-type mice treated with INO-1001 or vehicle. R6/2 mice treated with vehicle had a significantly reduced pCREB level compared to the vehicle treated wild type group (*P*<0.0001). pCREB levels were higher in R6/2 animals treated with INO-1001 compared to the vehicle treated R6/2 animals (*P*<0.0001).

As illustrated in Fig 8A–8L, our dual labeling study showed that INO-1001 increased pCREB immunoreaction product in the surviving spiny neurons of R6/2 mice. The intensity of pCREB, expressed in arbitrary units, was significantly lower in the saline treated R6/2 compared to the wild type littermates with a genotype effect F(1,3306) = 72.46 P<0,0001. Moreover, pCREB immunoreactivity was significantly more intense in INO-1001 treated R6/2 compared to both the saline R6/2 and the wild-type animals with a treatment effect F(1,3306) = 451.8 P<0,0001 and a genotype X treatment interaction F(1,3306) = 451.8 P<0,0001 as shown in Fig 8M.

#### 4) Analysis of BDNF

**Fig 9:**
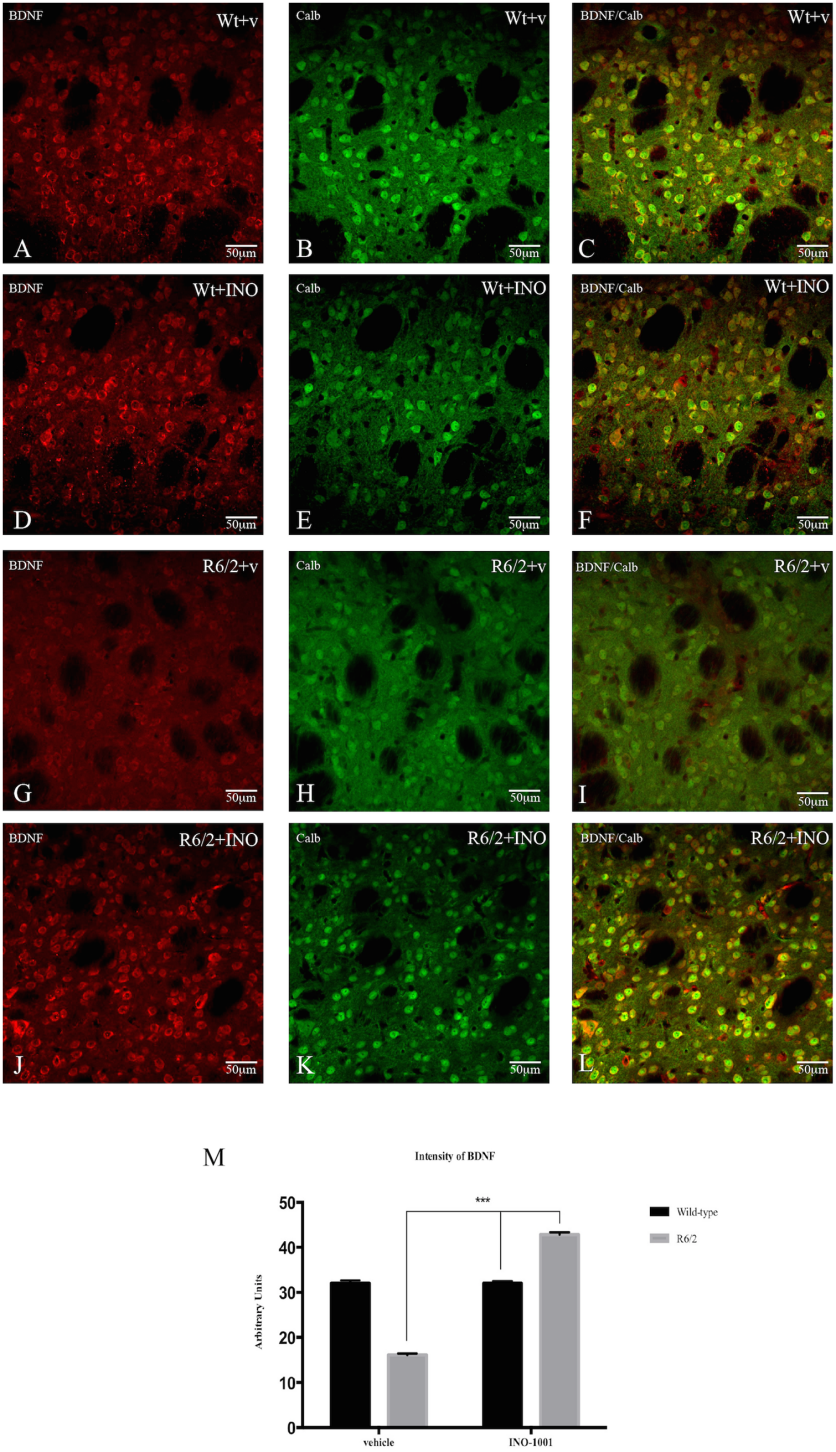
Effects of INO-1001 treatment on BDNF. Representative confocal laser scanning microscopy images of dual label immunofluorescence for CALB (green) and BDNF (red) in striatal samples from a vehicle treated wild type (**A-B-C**), INO-1001 treated wild type (**D-E-F**), vehicle treated R6/2 (**G-H-I**) or R6/2 treated with INO-1001(**J-K-L**). In (**M**) Quantification of the intensity of BDNF immunoreactivity associated with CALB-labeled striatal neurons. A two way ANOVA indicated a significant effect of genotype, treatment and genotype X treatment interaction (please see text). Data were analyzed with GraphPad Prism statistical software 6.0e using two-way ANOVA followed by a Tukey’s multiple comparison test. Tukey’s multiple comparison test indicated that there was no statistically significant difference in BDNF level wild-type mice treated with INO-1001 or vehicle. R6/2 mice treated with vehicle had a significantly reduced BDNF level compared to the vehicle treated wild type group (*P*<0.0001). Also in this case, BDNF levels were statistically higher in R6/2 animals treated with INO-1001 compared to vehicle treated R6/2 animals (*P*<0.0001).

We investigated the effect of treatment with INO-1001 on BDNF protein expression in the striatum of R6/2 mice. This observation was particularly relevant as BDNF is a CREB target gene. Therefore, we aimed at verifying if the anticipated increase in pCREB would lead to an up regulation of BDNF. As shown in Fig 9A–9L, INO-1001 was able to increase BDNF immunoreactivity in the R6/2 mice. Indeed, as shown in Fig 9M, a Two-way ANOVA analysis indicates a significant effect of genotype F (1, 4753) = 18.87816; P<0, 0001. Moreover, INO-1001 was effective in augmenting the protein expression of BDNF in the treated group compared to the saline–treated R6/2 mice with a treatment effect F (1, 4753) = 536.3125; P<0, 0001 and genotype X treatment interaction F (1, 4753) = 536.3125; P<0.0001.

#### 5) iNOS expression

Immunofluorescence showed enhanced iNOS immunoreactivity in R6/2 vehicle-treated mice, compared to INO-1001 treated R6/2 and vehicle- or INO-1001 treated Wt mice. These results confirmed that inhibition of PARP1 reduced the expression of some inducible genes, such as iNOS (S1 Fig).

### Western blotting

#### Expression of BCL2 and BAX

We examined the expression of the markers of apoptosis Bcl-2 and Bax in INO-1001 treated R6/2 mice. Protein levels of the antiapoptotic Bcl-2 (S2A and S2B Fig) were higher in the INO-1001 injected mice, while the pro-apoptotic factor BAX immunolabeling (S2A and S2C Fig) was higher in vehicle treated R6/2 mice. The Bax/Bcl-2 protein ratio was significantly decreased after INO-1001 treatment (*P*<0.001) (S2D Fig). These results suggest that INO-1001 treatment may positively shift Bax/Bcl-2 protein ratio and thereby facilitate neuronal survival.

## Discussion

The present data show that PARP inhibition by INO-1001 exerts a beneficial effect on the R6/2 mouse model of HD in terms of survival, neurological impairment and neuroprotection. These effects in promoting neurological recovery demonstrate that the PARP inhibitor used in this study is effective in suppressing PARP activation caused by HD degeneration.

Our study demonstrates the regulation of CREB activity, BDNF, and microglia activation all participate in the neurorescue activity of PARP inhibition by INO-1001. Moreover, we show that INO-1001 has a positive effect in behavioral aspects such as Rotarod performance and open field.

Activation of PARP-1 has been shown to be involved in the necrotic cell death occurring in response to excessive DNA damage under several pathological conditions [57].

In our study, we show that pCREB levels are increased in the striatum of R6/2 mice treated with INO-1001. A previous study by Wang and coworkers [58] described that PARP 1 activation stimulated by NGF administration is associated with an increase in CREB, and that the PARP 1 inhibitor 3-AB causes a decrease in CREB phosphorylation, in the hippocampus. These results are apparently conflicting with our finding of increased pCREB after PARP inhibition. On the other hand, Khodagholi and Ashabi [59] showed that salvia sahenica increases phosphorylated CREB in a rat Aß model of Alzheimer’s disease, and that this effect is associated with reduced apoptosis and decreased PARP 1 activity.

In our study, PARP inhibition promoted cell survival and was associated with an upregulation of CREB. CREB induces transcription of about 4000 target genes, including genes regulating apoptosis [60, 61]. Interestingly, while CREB phosphorylation promotes an increase in BDNF [62], we have previously shown that BDNF administration, possibly by a positive feedback mechanism, results in an increased CREB phosphorylation [63]. PARP 1 inhibition reduced apoptosis, as confirmed by our study, and was associated with a modulation of BAX and BCL-2 expression. We speculate that the effect on apoptosis results in increased pCREB levels, which in turn upregulates downstream survival factors such as BDNF. In a recent study [64], the decreased apoptosis observed in a model of Alzheimer’s disease was associated with an increase of pCREB.

We interpret the increase in CREB phosphorylation observed in our study to be, at least in part, mediated by increased cell survival and a decreased apoptosis.

Indeed, although not fully demonstrated, there is general agreement on the fact that apoptosis may be involved in several neurodegenerative disorders [65]. We show that inhibition of PARP 1 is neuroprotective for the striatum in the R6/2 model of HD, and this neuroprotective effect might be due to a number of factors.

We have observed an up regulation of BDNF in the striatal neurons. The mechanism(s) whereby PARP 1 inhibition increases striatal BDNF levels, and ameliorates its pathology are obviously speculative. BDNF is delivered to striatum by anterograde transport from cortical and, to a much lesser extent, midbrain striatal inputs [66, 67, 68]. However, retrograde transport of BDNF from striatum back to cortex is also proposed as a significant element of trophic support to the source cortical neurons. We suggest that the amelioration of striatal pathology by PARP 1 inhibition may reside in the maintenance of corticostriatal synaptic interconnectivity, with the secondary effect of maintaining retrograde transport of BDNF to cortex. Moreover, BDNF is a CREB-target gene; therefore, an increase in activated CREB could directly result in an increase in BDNF [69]. However, it is also possible that there is a direct effect of INO 1001 treatment on cortical CREB phosphorylation and BDNF synthesis resulting from the inhibition of neuroinflammation.

Moreover, we can speculate that PARP1 inhibition leads to activation NAD-dependent deacetylase sirtuin-1 (Sirt1) that would result in the increased level of BDNF via Akt modulation [70]. In this light, autophagy can be involved as Sirt 1 activation is able to rescue from polyglutamine induced toxicity through a modulation of autophagy, as described by Shin and coworkers [71]. Indeed, autophagy is the process of degradation of cytoplasmic proteins or organelles, and it eliminates toxic polyQ aggregates thereby reducing their cytotoxicity [72,73]. Because Sirt1 was shown to induce autophagy by deacetylating key autophagy-related proteins [74], we can to hypothesize that, in our model, increased Sirt1 activity due to PARP1 inhibition may induce the autophagy-mediated elimination of polyQ aggregates, thereby ameliorating their detrimental effect on the neurons of the R6/2 mice striatum.

It was previously demonstrated that PARP-1 deficient mice are protected against several pathological conditions such as myocardial infarction, streptozotocin-induced diabetes, LPS-induced septic shock, collagen-induced arthritis etc., suggesting that PARP-1 is implicated in inflammatory disorders [75].

We have also described a decreased number of activated microglia. The role of microglia merits attention. A supportive role for microglia in brain neuroplasticity stimulation possibly through BDNF production has been described in an animal model of brain ischemia [38]. In our study, we found that BDNF levels were increased in the surviving striatal neurons, and that microglia was reduced at the same time. Activated microglia has different effects depending on the nature of the stimulus, and also other factors, such as timing [76, 77, 78]. Neurodegeneration, as seen in the traumatic brain injury model (TBI), can arise from chronic activation of microglia and neuroinflammation [79].

We can also speculate that the potentially beneficial effect of microglia on BDNF production was not very marked in our model, where the detrimental effects on neuropathology were preponderant. Moreover, in the rat TBI model, PARP-1 inhibition by INO-1001 was able to reduce microglial activation [80]. Our data seem to be consistent with this specific effect of PARP inhibition.

Histopathological manifestations of neuroinflammation in brain have been described in many neurodegenerative diseases, including HD [81]. It has not yet been elucidated whether mutant huntingtin mediates neuroinflammation directly, and whether such phenomenon occurs primarily or in response to neuronal damage. Young and co-workers [82] have recently demonstrated that doubling the mutant huntingtin gene dose exacerbates the expression of neuroinflammatory markers.

In the R6/2 model of HD that we used in this study, neuronal loss was associated with microglial activation, and INO-1001 efficiently suppressed both phenomena (microglia activation and neuronal loss). This result suggests that the INO-1001 preserved neuronal survival, at least in part, by suppressing microglial activation; however, other mechanisms might play a role in the neurorescue. One other possible mechanism is the direct neuroprotection by INO-1001. Indeed, as shown by previous studies, INO-1001 can interrupt a programmed cell death pathway triggered by DNA damage, independently of its anti-inflammatory effects [83, 84].

Hamada and coworkers have reported that nitric oxide (NO) produced by inducible nitric oxide synthase (iNOS) is neurotoxic [85]. Other authors showed iNOS protein expression in microglial cells, neurons, and astroglial cells after acute spinal cord injury (SCI) [86]. In that study, inhibition of iNOS induced a reduction of apoptosis. Indeed, it is known that iNOS is able to induce the extrinsic apoptotic pathway that brings about caspase-3 activation and modulates apoptosis [86]. In endotoxin-stimulated glial cells, the expression of inducible nitric oxide synthase (iNOS) was reduced in the absence of PARP-1[87]. Our study is in agreement with these findings. Indeed, we showed a high expression of iNOS in striatal tissue of vehicle-treated R6/2 mice, where apoptosis is intense, and, in contrast, a decreased expression of iNOS in INO-1001 treated R6/2 mice. This confirms that INO-1001 reduces the activity of PARP-1, and that it counteracts apoptosis, as it was also confirmed by decreased expression of BAX in INO-100-treated R6/2 mice.

Neuroprotection achieved by INO-1001 can thus be explained by a number of mechanisms. An inhibitory effect on apoptotic death is most likely to occur, as demonstrated by the modulation of BAX and Bcl-2 in our model. Moreover, the inhibition of microglia as a mediator of neuroinflammation can be also responsible for the neurorescue operated by PARP inhibition [79]. PARP inhibition can also account for the Sirt 1-mediated BDNF upregulation [71], which translates in a protective effect for the striatal neurons. Finally, Sirt-1 might also mediate an autophagy process that eliminates toxic polyQ aggregates [74].

The sum of our data indicates that PARP inhibition can be a useful approach to reduce neurodegeneration and ameliorate symptoms in HD. Further studies are necessary to better understand the mechanisms of such powerful therapeutic agent.

## Supporting Information

S1 Fig Effects of INO-1001 treatment on iNOS expression. Representative confocal laser scanning microscopy images of immunofluorescence for Neurotrace (visualized by green fluorescence) and iNOS antibody (visualized by red fluorescence) in striatal tissue from a vehicle treated wild type (**A-B-C**), INO-1001 treated wild type (**D-E-F**), R6/2 vehicle-treated (**G-H-I**) and INO-1001 treated R6/2 mice (**J-K-L**).
(TIF)

S2 Fig Effects of Ino-1001 administration on Bax and Bcl-2 protein expression in R6/2 mouse brains. Western blot analysis of Bax and Bcl-2 proteins was performed in R6/2 and control mouse brains. In A: panel shows the representative immunoblots obtained for Bcl-2 and Bax at the expected size of 30 kDa and 21 kDa, respectively. The figure describes the increase of Bcl-2 in mice treated with INO 1001, and the upregulation of Bax in R62 mice treated with vehicle. B and C show quantitative data obtained from 4 separate experiments. The bottom panel (D) shows the Bax-to-Bcl-2 ratio in mice brains. Data are expressed as mean ± SEM. **P<0.001 vs. control.
(TIFF)
