# Supplementary figures and images for: PARP-1 Inhibition Is Neuroprotective in the R6/2 Mouse Model of Huntington’s Disease
Source: PLoS One. 2015 Aug 7;10(8):e0134482. doi: 10.1371/journal.pone.0134482 (PMC4529170; doi:10.1371/journal.pone.0134482)

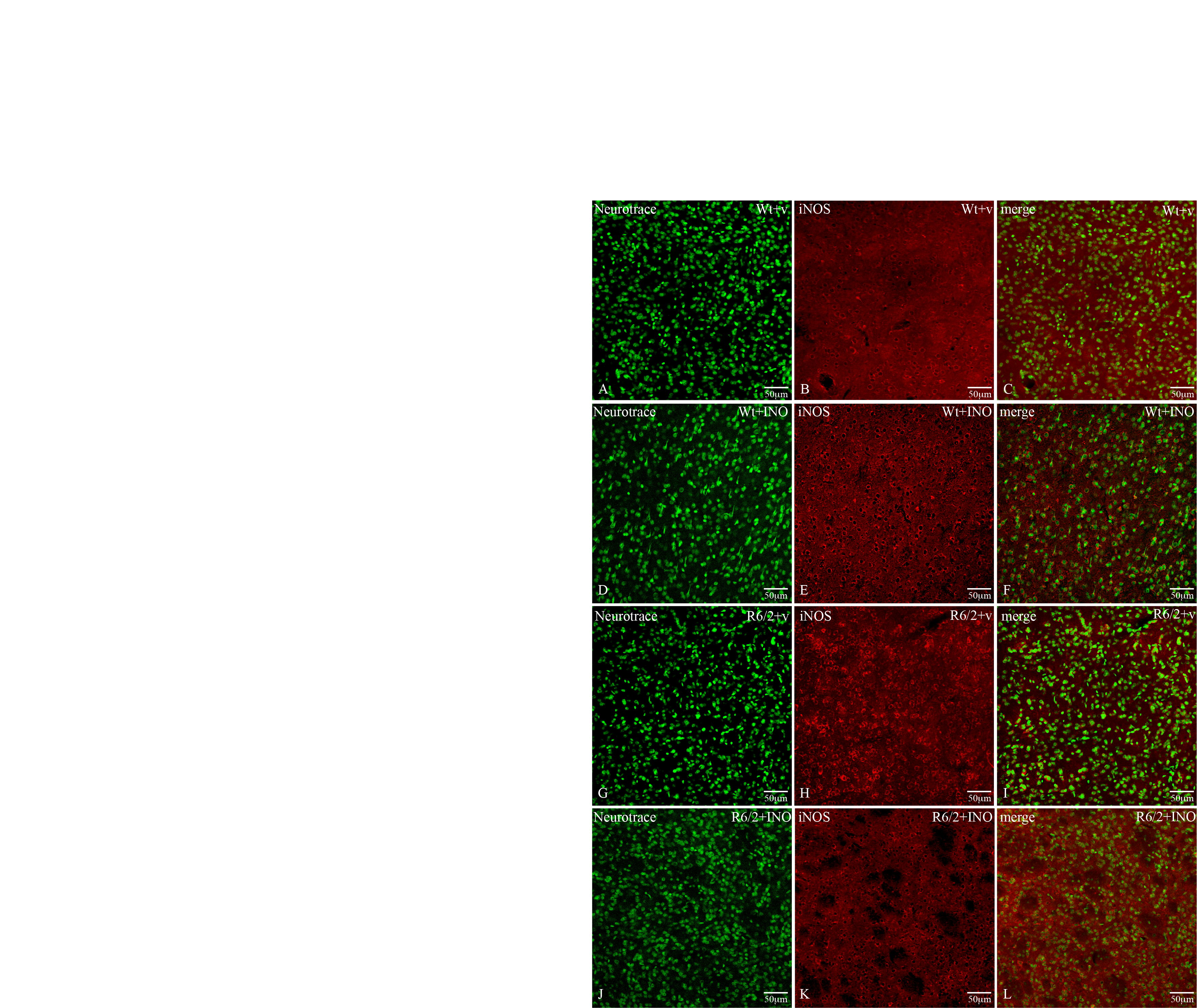

Supplement: S1 Fig — Representative confocal laser scanning microscopy images of immunofluorescence for Neurotrace (visualized by green fluorescence) and iNOS antibody (visualized by red fluorescence) in striatal tissue from a vehicle treated wild type (A-B-C), INO-1001 treated wild type (D-E-F), R6/2 vehicle-treated (G-H-I) and INO-1001 treated R6/2 mice (J-K-L). (TIF) [file pone.0134482.s001.tif]

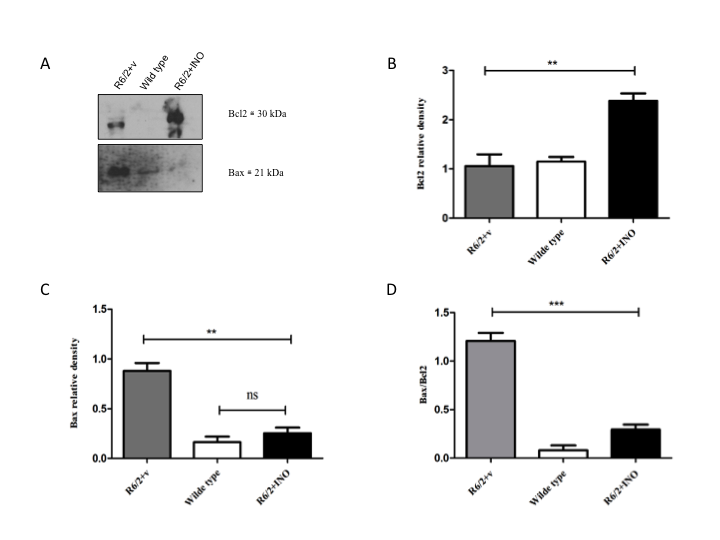

Supplement: S2 Fig — Western blot analysis of Bax and Bcl-2 proteins was performed in R6/2 and control mouse brains. In A: panel shows the representative immunoblots obtained for Bcl-2 and Bax at the expected size of 30 kDa and 21 kDa, respectively. The figure describes the increase of Bcl-2 in mice treated with INO 1001, and the upregulation of Bax in R62 mice treated with vehicle. B and C show quantitative data obtained from 4 separate experiments. The bottom panel (D) shows the Bax-to-Bcl-2 ratio in mice brains. Data are expressed as mean ± SEM. **P<0.001 vs. control. (TIFF) [file pone.0134482.s002.tiff]
